# Surgical treatment for patients with hemophilic pseudotumor-related femoral fracture: a retrospective study

**DOI:** 10.1186/s13018-021-02426-1

**Published:** 2021-04-21

**Authors:** Keyu Chen, Guiyong Jiang, Yaowen Xu, Yunping Yang, Zexiong Mao, Jiaxin Lv, Fei Liu, Bin Chen

**Affiliations:** 1 Division of Orthopaedics and Traumatology, Department of Orthopaedics, Nanfang Hospital, Southern Medical University, Guangzhou, China; 2 Department of Health Management, Nanfang Hospital, Southern Medical University, Guangzhou, China

**Keywords:** Hemophilia, Pseudotumor, Femoral fracture, Reconstruction protocol

## Abstract

**Background:**

Hemophilic pseudotumor (HPT)-related fracture is a rare but severe complication in patients with HPTs. These fractures often occur in femurs. There is no consensus on the standard surgical protocol for HPT-related femoral fracture. The present retrospective study evaluated the outcomes of these patients treated with surgical interventions.

**Methods:**

Ten patients with HPT-related femoral fractures who were treated with 14 surgical procedures due to 11 fractures in our hospital from January 2014 to April 2020 were evaluated retrospectively. Demographic data, fracture location, complications after surgery, and follow-up outcomes were recorded and analyzed. The mean follow-up period was 39.7 months.

**Results:**

The mean age at surgery was 31 years. Closed reduction external fixation (CREF) was originally performed in 2 patients, open reduction internal fixation (ORIF) was performed in 4 patients, screw fixation alone was performed in 1 patient, brace immobilization was performed in 1 patient, and amputation was performed in 3 patients. Bone union was observed in 5 patients, and an adequate callus was visible in 2 patients. Both patients with CREF had pin infections. Nonunion combined with external fixation (EF) failure occurred in 1 patient, and the plate was broken after ORIF. Three patients underwent autogenous or allogeneic cortical strut grafting. Three patients had HPT recurrence.

**Conclusions:**

It is necessary to perform surgery in patients with HPT-related femoral fractures. Surgical treatments must consider fracture stabilization and HPT resection. Internal fixation is preferable, and EF should only be used for temporary fixation. If the HPT erodes more than one third of the bone diameter, strut grafts are necessary for mechanical stability. Amputation is an appropriate curative method in certain situations.

## Introduction

Hemophilia is an X-linked hereditary bleeding disorder caused by a deficiency or lack of coagulation factor VIII (FVIII), which leads to hemophilia A (HA), or factor IX (FIX), which leads to hemophilia B (HB) [1]. This deficiency results in recurrent bleeding episodes in the musculoskeletal system. Hemophilic pseudotumor (HPT) results from recurrent bleeding from extra-articular bone or soft tissues, and it develops in severe hemophilia with a prevalence of 1–2% [2, 3]. HPTs generally progress asymptomatically for years and cause symptoms when pathological bone fracture or neurovascular compression occurs [4]. Gilbert described two clinical types, proximal pseudotumors and distal pseudotumors. Proximal pseudotumors occur in the proximal skeleton of adult patients, especially around the femur and pelvis, develop slowly and do not respond to conservative treatment. Distal pseudotumors occur distal to the wrist and ankle, develop rapidly, and are primarily seen in children and adolescents [4, 5]. HPTs are classified into 3 types according to the imaging features and location: soft tissue, subperiosteal, and intraosseous types [6]. HPTs normally involve adjacent bone and result in massive bone destruction [7].

Patients with hemophilia often have osteoporosis or low bone density [8]. Pathological fractures are more likely to occur in hemophilia patients with HPTs, and these fractures are caused by minimal trauma or have no obvious cause. The femur is the most susceptible long bone to HPTs, and most HPT-related pathological fractures occur in this bone [9–11].

Surgery is an effective treatment for HPTs, especially cases complicated by HPT-related fractures. However, there is no consensus on the standard surgical protocol because of the complexity and variety of HPTs and fractures. Surgeons may encounter many challenges during surgery, such as abnormal anatomy, multiple fixation options, and bone reconstruction considerations. Complications, including nonunion, infection, fixation failure, inhibitor development, and pseudotumor recurrence, should not be ignored because they exert a profound influence on patient outcomes [7, 12]. Avoiding complications is challenging for surgeons, and sometimes these complications are difficult to resolve. Due to the rarity of these fractures, reports of surgical treatment for patients with HPT-related femoral fractures are scarce. The present retrospective study pooled our experience to evaluate the outcomes of orthopedic surgery for patients with HPT-related femoral fractures.

## Materials and methods

We retrospectively reviewed patients with HPT-related femoral fractures who underwent orthopedic surgery in our hospital between January 2014 and April 2020. Patients with conservative treatment, HPT-mimicking tumors or defective medical records were excluded from the study. Hemophilia was classified as severe (< 1%), moderate (1~5%), or mild (>5%) depending on the level of FVIII/IX activity.

The units of coagulation factor were dynamically adjusted according to the concentration of factors during the perioperative period. The perioperative factor replacement protocol was based on literatures and our experience [13, 14]. Patients with hemophilia A received plasma-derived or recombinant FVIII throughout the entire treatment phase. Patients with hemophilia B received recombinant FIX only on the day of surgery, and they received prothrombin complex concentrate subsequently due to economic considerations. All patients received coagulation factor replacement preoperatively to maintain a peak factor level of over 100% during surgery, a factor level of 80% on postoperative days 1–2, 40–60% on postoperative days 3–6, and 30–40% on postoperative days 7–14. A factor level of 20–30% was maintained until the end of the rehabilitation period.

The same surgical team performed all surgical procedures. For HPT excision, a single incision was made to reduce the fractures and expose the pseudotumors. Pseudotumor resection began with as much normal tissue as possible. If the pseudotumors were adjacent to nerves and blood vessels, the surrounding cyst wall was only partly incised, and the fibrosed tissue and clotted blood contained within the cyst were drained. The remaining wall was closed with sutures. The cyst cavity within the soft tissue was eliminated using a biodegradable gelatin sponge and hemostatic gauze, and the bone cavity was filled with autologous or allogeneic cancellous bone grafts combined with manual compression of the grafts into the cavity. Elastic bandages were used to wrap the affected area with compression for 2 weeks. HPT curettage and bone grafting were performed for cystic lesions originating within the bone. If a massively eroded unilateral cortex was present, an allogeneic or autogenous bone strut graft was used for mechanical stability. For fracture management, closed reduction external fixation (CREF), open reduction internal fixation (ORIF), screw fixation, brace immobilization, and amputation were performed according to the fracture situation and patients’ desires. All patients or patients’ parents provided informed consent for the use of their anonymous data for research purposes, and the Ethics Committee of our hospital approved the study.

## Results

Four of the 14 patients were excluded from the study because they were treated conservatively. Therefore, data from 10 patients were available for the study, including 7 with HA and 3 with HB; 14 surgical procedures were performed due to 11 fractures. All patients were male, with an average age of 31 years (11 to 46 years). The mean follow-up period was 39.7 months (6 to 82 months). Five patients were classified into the severe group, and 5 patients were classified into the moderate group. Four fractures were caused by falling, and the other fractures had no obvious cause. Table 1 shows the general characteristics of the patients.

**Table 1:** General characteristics of the patients

Patient (No.)	Age	Diagnosis	Severity	Fracture location	Type of treatment	Follow-up (months)	Outcome
1	46	HA	Severe	L shaft	CREF	6	Pin infection after 3 months combined with visible adequate callus, and inhibitor developed after 4 months; patient refused further intervention. Died due to severe bleeding and infection 6 months later
2	23	HA	Severe	L distal	HPT excision, ORIF	83	Bone union, no HPT recurrence
3	44	HA	Moderate	R shaft	CREF	61	Pin infection after 2 weeks, treated with dressing change and discontinuous use of antibiotics, was not resolved until fixation failure. Fixation failed with fracture nonunion after 13 months
				R shaft (revision)	HPT excision, ORIF		HPT recurrence, plate broken 3 months after ORIF
				R shaft	High-thigh amputation		No HPT recurrence
4	20	HA	Moderate	R shaft	HPT excision, ORIF, allogeneic cortical strut graft	30	Bone union, no HPT recurrence
5	31	HB	Severe	L distal	HPT excision, ORIF, allogeneic cortical strut graft	31	Bone union, no HPT recurrence
6	38	HA	Severe	L shaft	High-thigh amputation	45	No HPT recurrence
7	26	HB	Moderate	L shaft	Left hip disarticulation, HPT excision in the right thigh	77	HPT recurrence after 3 months in the right thigh, femoral fracture caused by HPT compression after 4 years
				R shaft	R high-thigh amputation		No HPT recurrence
8	11	HB	Moderate	R distal	HPT curettage, allogeneic cancellous bone graft, immobilization with brace	27	Bone union, no HPT recurrence
9	28	HA	Severe	R distal	HPT excision, autogenous cortical strut bone graft, allogeneic cortical strut bone graft, screw fixation alone	29	Bone union, thigh HPT recurrence after 1 year was treated with coagulation factor replacement without further progress. Tibial shaft fracture resulted from trauma 1 year later, was treated with ORIF
10	48	HA	Moderate	R shaft	HPT excision, ORIF	8	Visible adequate callus was observed, no HPT recurrence

*HA* hemophilia A, *HB* hemophilia B, *HPT* hemophilic pseudotumor, *ORIF* open reduction internal fixation, *CREF* closed reduction external fixation, *R* right, *L* left

The mean operative time was 195.5 min (82 to 268 min). The median amount of intraoperative blood loss was 450 ml (10 to 1200 ml). The mean hospital stay was 21.9 days (9 to 32 days). One patient developed postoperative hematoma, which was resolved using ultrasound-guided puncture and drainage. One patient had abnormal postoperative wound aseptic exudation, which was resolved by dressing changes.

**Fig. 1:**
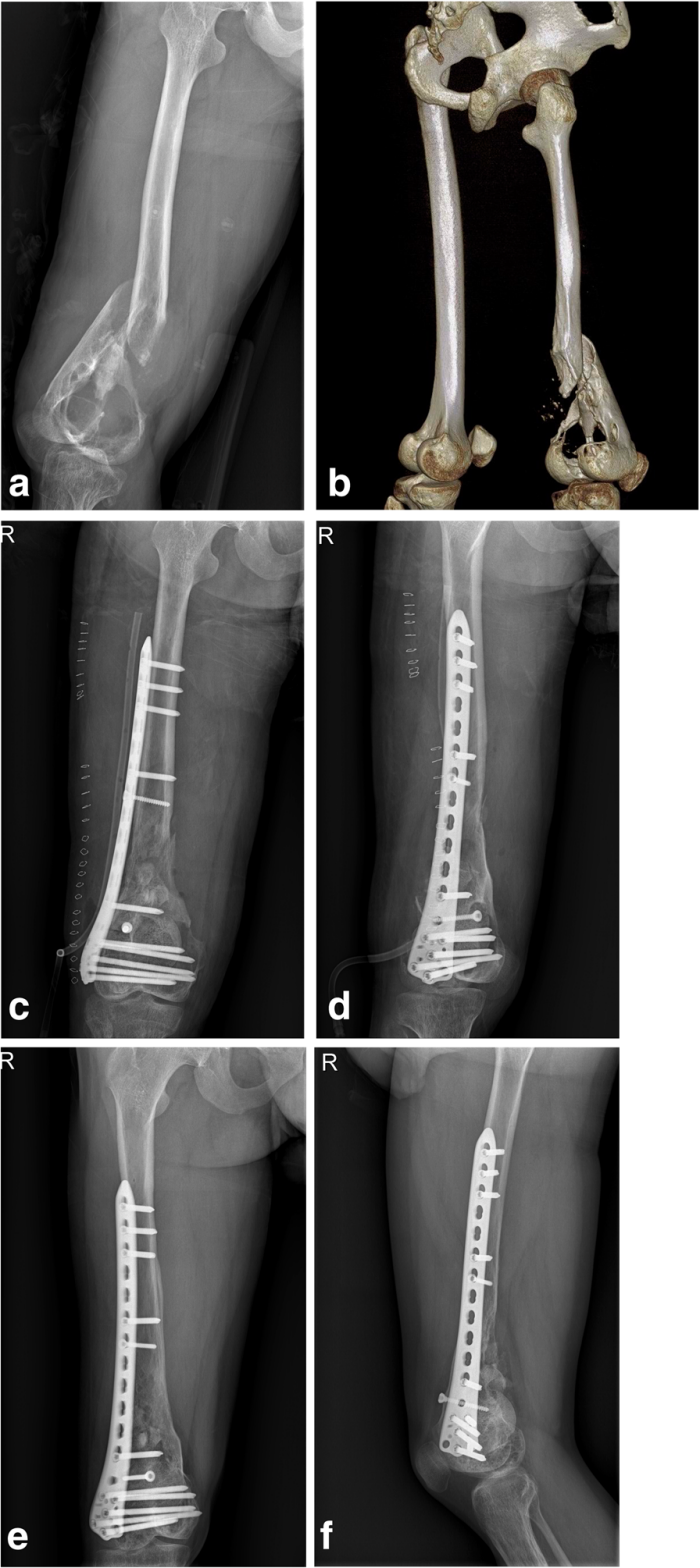
Surgical treatment with locking plate and allogeneic cortical strut graft was applied to a 23-year-old man (case 4) with a right distal femoral HPT-related fracture. Radiographs were taken at the **a**, **b** preoperative, **c**, **d** postoperative, and **e**, **f** 20-month postoperative timepoints

Seven fractures occurred in the femoral shaft, and the other 4 fractures occurred in the distal femur. For fracture management, CREF was originally performed in 2 patients, ORIF was performed in 4 patients, screw fixation alone was performed in 1 patient, brace immobilization was performed in 1 patient, and amputation was performed in 3 patients. All patients treated with ORIF underwent fixation with anatomically locking plates. Three patients underwent autogenous or allogeneic cortical strut graft implantation. Grafts included allogeneic/autogenic fibula and allogeneic femoral head (Fig. 1). Bone union was observed in 5 fractures, and adequate callus was visible in 2 fractures.

**Fig. 2:**
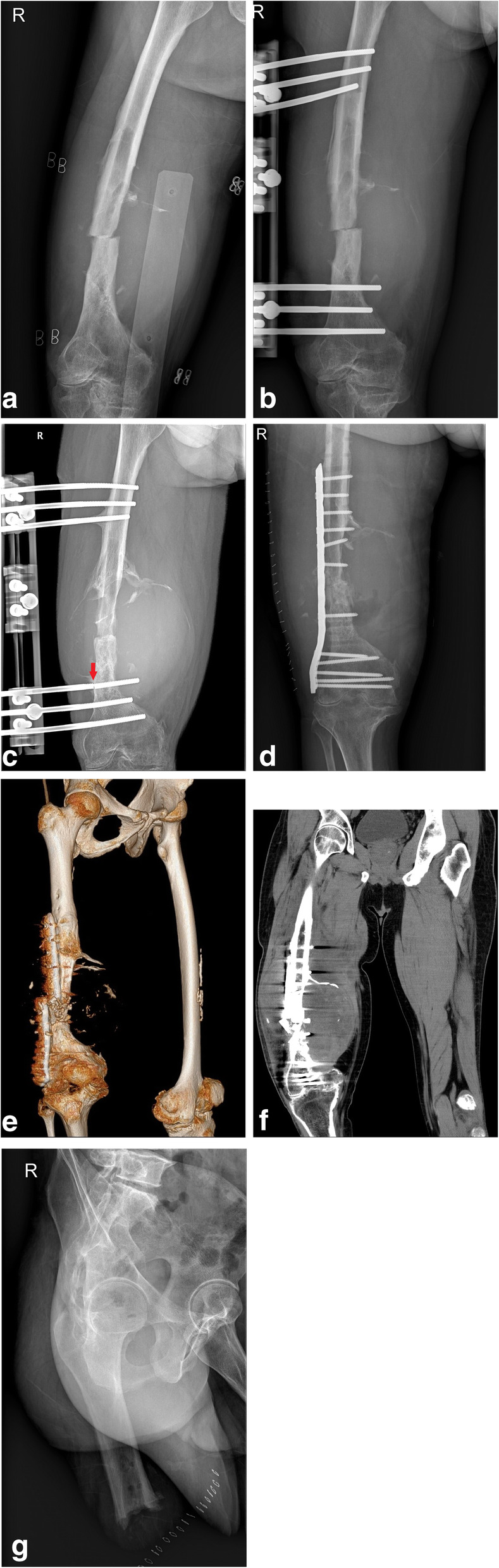
Surgical treatment with CREF to a 46-year-old man (case 2) with a right femoral shaft HPT-related fracture. **a**, **b** Preoperative and postoperative radiograph of CREF. **c** Bone nonunion combined with fixator failure (red arrow) 13 months later. **d** ORIF performed and HPT excision 14 months later. **e**, **f** HPT recurrence and plate broken 6 months after ORIF. **g** Amputation performed finally

Both patients treated with CREF had pin infections. Although an adequate callus was visible in 1 patient, he developed high titer coagulation inhibitor, refused further intervention, and died due to severe bleeding and infection 6 months after surgery. The other patient was treated with dressing changes and the discontinuous use of antibiotics, and the infection was not resolved until fixation failure occurred. In this patient, fixator failed combined with fracture nonunion after 13 months, and the patient underwent revision surgery of HPT excision and ORIF. However, the plate was broken, and HPT recurred 3 months later. High-thigh amputation was performed to ultimately resolve the symptoms (Fig. 2).

Three patients had recurrent pseudotumors. Except for the one patient mentioned above, one other patient had a recurrent pseudotumor in the other thigh 3 months after excision, and pseudotumor progression and bone erosion occurred, which resulted in another femoral fracture 4 years later. The other patient had HPT recurrence 1 year after the primary surgery, and HPT progression was halted with the use of coagulation factor replacement therapy.

## Discussion

Poor musculature and reduced bone mineral density may predispose patients with hemophilia to the risk of fractures [9]. HPT-related fractures are a severe complication of HPTs and may be caused by minimal trauma or have no obvious cause [15]. Jensen et al. demonstrated that bone may be affected by HPTs via pressure necrosis, and the femur is the most common site of involvement [11]. HPTs generally progress asymptomatically until pathological bone fracture or neurovascular compression occurs [4]. The literature recommends surgical treatment for HPTs combined with bone erosion or fracture [4, 12]. There is no consensus on the standard surgical protocol because of the complexity and variety of HPTs and fractures. Therefore, surgery is very challenging for surgeons. Compared with other studies [12, 15], this work mainly discussed the fixation and limb construction options in patients with HPT-related femoral fractures.

Seven patients received internal fixation: 1 with screws and 6 with plates. HPT recurrence caused plate breakage in 1 patient, who eventually underwent amputation. Bone union was observed in 5 patients, and visible adequate callus was observed in 1 patient. Intramedullary nails are the preferable fixation method for HPTs combined with fractures [13], but a locking plate was used in the most patients in our study because the HPT resection, fracture reduction, and internal fixation may be completed with a single incision. Because pseudotumors generally erode the bone cortex, the extramedullary blood supply is affected during pseudotumor resection, and the reaming required for intramedullary fixation may affect the intramedullary blood supply [16], which aggravates the damage to the bony blood supply. Although studies demonstrated the effects of metal internal fixation on stress shielding, which cause peri-implant fractures and nonunion [12], rigid internal implants are necessary to provide support for the fractured bone and facilitate union at an appropriate position to allow patients to perform early functional exercises. The use of a long plate reduces the risk for fixation failure and spreads the stress to the entire bone [17, 18].

When the pseudotumor is large and difficult to remove or the bone is massively eroded, amputation may be the last option, but it is effective. The advantage of amputation is that it completely eliminates pseudotumors and reduces the cost of surgery and the risk of readmission. Amputees do not have psychological concerns, such as recurrence, failure of the osteosynthesis, and infection. When severe bone deconstruction occurs, pseudotumors almost completely erode the bone, and the affected limb loses its bony support, which makes it very difficult to reconstruct the limb. Buchowski et al. reported that reconstruction with a custom total femoral prosthesis was a valuable alternative to amputation in massive pseudotumors of the femur and soft tissues of the thigh [19]. However, the long-term outcome of custom total femoral prostheses is not clear, and complications are common [20]. Three patients with pathological fractures caused by pseudotumors ultimately chose amputation in our study. No HPT recurrence was observed in these patients. Due to the presence of pseudotumors, the surrounding tissues, including blood vessels and nerves, are anatomically abnormal, and there is a risk of excessive bleeding during amputation. Therefore, the surgery should be performed delicately to avoid iatrogenic vascular damage and excessive bleeding, especially when tourniquets are not applied. Patients in our study who underwent amputation generally used crutches to walk because of their poor economic situation. Attention should be paid to the additional bleeding risk of the upper limbs caused by the long-term use of crutches.

EF procedures are typically used for temporary fracture fixation, deformity correction, and limb lengthening. For fracture treatment, a major benefit of external fixators is that they stabilize a fracture without the need for open reduction or invasive surgery at the fracture site [21]. In theory, EF stabilizes the fracture in a minimally invasive manner and simultaneously avoids interfering with the pseudotumor. Two fracture patients in our study underwent CREF, and both developed pin infections. Similar to conservative treatment, EF does not fundamentally solve the cause of the fracture, i.e., EF does not remove the pseudotumor. Even when the fractures are stabilized, it is difficult to prevent HPT progression using conservative treatment, and uncontrolled progression will affect the pins and bone, resulting in pin infection, bleeding, and bone erosion. To avoid pseudotumors, the entry points of the pins are located far from the fracture site, which means that fixation may not be sufficiently strong to control fracture displacement. Therefore, we do not recommend EF for patients with HPT-related fractures. However, when a patient’s overall condition is not suitable for open surgery or the condition of the soft tissue is poor, EF may provide temporary fixation. After the situation is corrected, the external fixation should be replaced with an internal fixation.

For HPT-related fractures, the main purpose of surgery is to remove the pseudotumors while providing stable conditions for bone union. Zhai et al. reported the use of structural bone grafts for bone defects > 5 cm caused by HPTs [12]. We used structural internal fixation with bone graft for patients with massive bone defects, and graft incorporation was observed in all patients. When autologous grafts are not sufficient, allogeneic structural bone may also be used. Majoor et al. used allogeneic strut bone grafts for the treatment of fibrous dysplasia of the proximal femur with a mean follow-up of 13 years. They argued that cortical allografts were less prone to pathological fibrous dysplasia of bone, and therefore, less prone to resorption and failure [22].

For patients without actual fracture, we recommend strut grafts for mechanical stability when the HPT has eroded more than one third of the bone diameter. However, whether prophylactic internal fixation is performed should be based on radiographic and intraoperative findings according to the surgeon’s decision. Mirels analyzed 78 metastatic long bone lesions from 28 patients, and the results showed that the rate of fracture significantly increased when the size of the lesion was more than two thirds of the bone diameter [23].

There are some limitations in this study that should be noted. This study had a retrospective design and involved only a small number of patients. Because the patients in this study were from different cities, it sometimes took several months for them to return to the hospital for review, which made it difficult for us to accurately and dynamically evaluate the situation with regard to bone union. Our follow-up period was relatively short.

## Conclusions

Surgery is necessary in patients with HPT-related femoral fractures. Surgical treatments must consider fracture stabilization and HPT resection. Internal fixation, especially the plate, is preferable, and EF should only be used for temporary fixation. Strut grafts are necessary for mechanical stability when the HPT erodes more than one third of the bone diameter. Amputation is a suitable surgical option when a pseudotumor is large and difficult to remove and bone reconstruction is complex.

## Data Availability

The datasets used and/or analyzed during the current study are available from the corresponding author on reasonable request.

## Abbreviations

FVIII: Coagulation factor VIII
FIX: Coagulation factor IX
HA: Hemophilia A
HB: Hemophilia B
HPT: Hemophilic pseudotumor
ORIF: Open reduction internal fixation
CREF: Closed reduction external fixation
CR: Closed reduction
